# Chemsex, Anxiety and Depression Among Gay, Bisexual and Other Men Who have Sex with Men Living with HIV

**DOI:** 10.1007/s10461-023-04041-z

**Published:** 2023-03-25

**Authors:** David James Field, John de Wit, Martin P. Davoren, Eilis J. O’Reilly, Chantal Den Daas

**Affiliations:** 1 Institute of Applied Health Sciences, University of Aberdeen, Aberdeen, Scotland; 2 Gay Men’s Health Service, Health Service Executive, Heytesbury Street, Dublin 8, Ireland; 3 Department of Interdisciplinary Social Science, Utrecht University, Utrecht, the Netherlands; 4 The Sexual Health Centre, Cork City, Cork, Ireland; 5 School of Public Health, University College Cork, Cork, Ireland

**Keywords:** MSM, Chemsex, Mental health, HIV, Anxiety, Depression

## Abstract

Chemsex is common among gay, bisexual and other men who have sex with men (gbMSM). Although not always categorised as problematic, a link with psychological distress has been reported and might be exacerbated amongst gbMSM living with HIV, as HIV has been associated with anxiety and depression. A cross-sectional online survey of gbMSM living with HIV (n = 359) was performed incorporating the Hospital Anxiety and Depression Scale and sociodemographic variables including, HIV characteristics, chemsex and sexual behaviours. Logistic regression analysis was used to find associations with anxiety or depression. Many participants engaged in chemsex (48.5%, n = 174). Chemsex was associated with lower odds of depression (aOR 0.45, 95% CI 0.23–0.85) and not associated with anxiety (aOR 0.66, CI 0.40–1.09). Although chemsex is a public health concern; we found it was associated with lower levels of depression in gbMSM living with HIV. However, causal inference is not possible, as gbMSM with higher levels of depression might engage in chemsex less.

## Introduction

HIV has become a chronic rather than life-threatening condition due to significant advances in treatment [1]. However, despite significant improvements in efforts to end the epidemic, critical issues for people living with HIV remain. Quality of life in people living with HIV is known to be reduced, and they experience higher levels of psychological distress, including anxiety and depression [2]. Reduced levels of quality of life might be exacerbated in specific subgroups. Indeed, gay and bisexual men who have sex with men (gbMSM) face poorer outcomes related to their mental health when compared to their heterosexual counterparts [3]. This sexual orientation-related disparity in mental health has been attributed to the Minority Stress Theory [4]. The Minority Stress Theory posits that actual and perceived stigma, fear of rejection, internalised homophobia and concealing one’s sexuality all contribute to a higher prevalence of psychological distress.

Another difference between gbMSM and their heterosexual counterparts, which may contribute to psychological distress, is engagement in chemsex [5]. Chemsex is defined as drug use before or during sex *with the specific intention to improve or prolong sex* and has been described as a significant public health concern [6]. Some of the substances commonly associated with chemsex include but are not limited to; mephedrone, crystal meth and GHB/GBL [7]. These substances can be used independently or in combination, known as polydrug use. Chemsex has been most commonly observed among gbMSM [8]. Research indicates that gbMSM may be as much as three times more likely to partake in chemsex than their heterosexual counterparts [8], with multiple studies indicating gbMSM living with HIV are more likely to engage in chemsex [8]. Chemsex has also been linked to other risk-related sexual behaviours, including; transactional sex, group sex and fisting [3]. Drugs often linked to chemsex have been associated with psychological distress [8] with one study indicating that 25% of gbMSM engaging in chemsex self-reporting that it negatively impacted their mental health [9]. It has also been previously stated that there were higher rates of anxiety and depression among gbMSM chemsex users [9].

Data from the European Men Who Have Sex with Men Internet Survey (EMIS) [10] highlighted Amsterdam as an urban centre where participants reported the highest prevalence of chemsex use in the 4 weeks before completing the survey (59%). This study also showed an association between chemsex and living with HIV. Participants living with HIV had almost five times greater odds of engaging in chemsex [9]. This might, however, be an overestimation, as EMIS participants, in general, were younger, and more sexually active compared to the general gbMSM population [11]. Nevertheless, subsequent research has found that 20% of gbMSM in the Netherlands have reported a desire to change their behaviours related to drug use in general and chemsex in particular [12].

A 2019 systematic review of chemsex use indicated the practice is associated with various negative psychosocial impacts, including loss of friends and employment and reduced mental health [8]. Although much research to date has focused on the adverse outcomes associated with chemsex, it should be noted that not all research links chemsex to adverse mental health outcomes. Australian research indicates that one of the most ubiquitous chemsex drugs: GHB, is not associated with experiencing anxiety or depression [13].

The present study analyses the data from the online “Survey of Men and Sexuality” (SMS) [14]. We aimed to assess the prevalence of and associations between chemsex, anxiety and depression for gbMSM living with HIV. Research indicates that gbMSM living with HIV are more likely to partake in chemsex [8], this may be a volatile combination that requires further investigation. While much has been written about the possible relationship between chemsex and mental health, this study focuses specifically on gbMSM living with HIV to better understand the associations between chemsex use and the likelihood of experiencing anxiety and depression in this key population who are more likely to experience reduced mental health [2].

## Methods

### Study Design and Participants

SMS was a cross-sectional online survey of gbMSM in the Netherlands. It ran from February 2018 to June 2018 and was available in six languages (Dutch, English, French, Farsi, Arabic and Turkish). Participants were recruited by self-inclusion via social media and gbMSM geo-location apps for dating and hook-ups. The inclusion criteria were being male, aged 16 years or older, living in the Netherlands and one of the following: (i) having sex with men, (ii) being attracted to men, or (iii) expecting to have sex with men in the future. The total number of participants who completed the SMS survey was 3921. For the purpose of this research, only gbMSM living with HIV were included (9.15%, n = 359) Data was collected anonymously and participants provided informed consent before commencing the survey. The Ethical Review Board of the Faculty of Social and Behavioural Sciences, Utrecht University approved this study (FETC17-131).

### Measures

The Hospital Anxiety and Depression Scale (HADS) was used to assess participant’s experiences of anxiety and depression [15]. This assessment tool was chosen as it allows for the self-assessment of clinically significant anxiety and depression symptoms. It has been used with people requiring inpatient care as well as people attending outpatient clinics, including receiving HIV care [16], The HADS has also been utilised outside of the clinical setting [16]. The scale contains a total of 14 items, including seven to assess for anxiety (HADS-A) and seven to assess for depression (HADS-D) [16]. A score of greater than seven is used to indicate clinically significant anxiety or depression [16]. Research has found good internal consistency using the HADS assessment tool across 18 studies [16] and the HADS was found to perform well when compared to other measures used to assess for anxiety and depression, including the General Health Questionnaire (GHQ) and the Beck Depression Inventory [16].

To assess for any drug use and engagement in chemsex, participants were asked if they had used any of the following substances before or during sex in the last 6 months with responses recoded as yes ever/no never responses from a 5-point scale (1 = never, 5 = always). Drugs assessed included; nitrites (poppers), cannabis, cocaine, nitrous oxide, Valium, ecstasy, amphetamine (speed), methamphetamine (crystal meth), mephedrone, y-hydroxybutyrate/y-butyrolactone (GHB/GBL), ketamine, LSD, methoxetamine, mushrooms, heroin and other designer drugs. To limit our research to drugs associated with chemsex, an 8-chem definition was used and defined by the use of one or more of the following substances; mephedrone, methamphetamine, GHB/GBL, cocaine, ketamine, speed, ecstasy/MDMA or other designer drugs [17–19]. Polydrug use indicated by the use of three or more of the drugs assessed, and could additionally include any use of erectile stimulants.

Sociodemographic variables assessed were age, education, sexual orientation and migration background. HIV characteristics included; number of years since HIV diagnosis (< 5 years or recent infection, 6–25 years or since the introduction of effective antiretroviral therapies and > 25 years or since before the introduction of antiretroviral therapies), HIV care attendance (yes/no), and viral load (detectable/undetectable). Sexual behaviours assessed were; the numbers of sexual partners, previous sexually transmitted infections (STIs) and group sex in the previous 6 months.

### Data Analysis

Descriptive statistics were calculated to characterise psychological distress and chemsex engagement. Univariable and multivariable logistic regression were performed to assess the relationships between anxiety and depression and chemsex use, demographic variables, HIV characteristics and sexual behaviours [20]. We checked for collinearity using the variance inflation factor. To build the multivariable models, backward stepwise multivariable logistic regression analyses were performed. This led to a reduced model with only associated variables reported [20]. Associations were examined using adjusted Odds Ratios (aOR) and 95% confidence intervals.

## Results

### Participant Characteristics

As described in Table 1, 67.4% (n = 242) of participants were aged over 40 (mean age = 45.62, SD = 12.02). The participants were highly educated, with 66.2% (n = 237) having attained tertiary or postgraduate level education, identified as gay (94.2%, n = 338), and were mostly of Dutch origin (77.7%, n = 279). Most participants (76.9%, n = 270) received their HIV diagnosis more than 5 but fewer than 25 years ago, meaning they were diagnosed after widespread availability of antiretroviral medications to treat HIV but before wide acceptance of the *undetectable equals untransmittable* message. Most were engaged in HIV care (96.1%, n = 345) and had an undetectable viral load (88.6%, n = 318). In the 6 months preceding the survey, over one-third of participants had more than ten sexual partners (36.5%, n = 131), and one-third were diagnosed with an STI (32.3%, n = 116).

**Table 1:** Participant demographics including socioeconomic demographics, HIV information, sexual behaviours (inc. risk factors) stratified for participants with anxiety and depression as identified by HADS scores

	Total population		Potential anxiety disorder		Potential clinical depression	
	N	%	N	%	N	%
Total	359	100	90	25.1%	62	17.3%
Socio demographic variables						
Age						
< 25	20	5.6	6	6.7	1	1.6
25–39	97	27.0	31	34.4	18	29.0
> 40	242	67.4	53	58.9	43	69.4
Education						
Second level or lower	121	33.8	36	40.0	23	37.1
Third level/postgraduate	237	66.2	54	60.0	39	62.9
Sexual orientation						
Gay	338	94.2	83	92.2	57	91.9
Bisexual & heterosexual MSM	21	5.9	7	7.8	5	8.1
Migration background						
Dutch	279	77.7	64	71.1	45	72.6
Non-Dutch, Western	28	7.8	6	6.7	5	8.1
Non-Dutch, non-Western	52	14.5	20	22.2	12	19.4
HIV characteristics						
Years since HIV diagnosis						
< 5 years (recent infection)	56	16.0	19	21.1	7	11.3
6–25 years (post ARVs)	270	76.9	67	74.4	52	83.9
> 25 years (pre ARVS)	25	7.1	4	4.4	3	4.8
On HIV treatment						
Yes	345	96.1	87	96.7	60	96.8
No	8	2.2	3	3.3	2	3.2
Viral load						
Undetectable	318	88.6	82	91.1	56	90.3
Detectable or unknown	27	7.5	8	8.8	6	9.7
Sexual practice						
Number of partners*						
0–1	74	20.6	25	28.1	20	32.8
2–5	86	24.0	25	28.1	15	24.6
6–10	67	18.7	15	16.7	8	13.1
> 10	131	36.5	24	26.7	18	29.5
Sexual behaviours*						
Sexually transmitted infection						
Yes	116	32.3	33	36.7	22	35.5
No	227	66.2	57	63.3	40	64.5
Group sex						
Yes	187	59.6	44	49.4	28	45.9
No	127	40.4	45	50.6	33	64.5
Polydrug use						
Yes	171	47.6	41	45.6	23	37.1
No	188	52.4	49	54.4	39	62.9
Chemsex** (8 chems)**						
Yes	174	48.5	43	47.8	23	37.1
No	185	51.5	47	52.2	39	62.9

*aOR* adjusted odds ratio, *ARVs* anti-retro viral medications, *CI* confidence interval, *Gbmsm* gay, bisexual and other men who have sex with men, *HIV* human immunodeficiency virus

*In the previous 6 months

**8 Chems: mephedrone, methamphetamine, GHB/GBL, cocaine, ketamine, speed, ecstasy/MDMA and/or other designer drugs

There was a high prevalence of drug use, with 48.5% (n = 174) of participants engaged in chemsex, and 47.6% (n = 171) of participants engaged in poly drug use in the 6 months preceding the survey.

The HADS identified 90 (25.1%) participants with a potential anxiety disorder, of whom 42 participants scored mild, 40 moderate, and 8 severe. Additionally, 62 participants had a potential clinical depression (17.3%), of whom 33 scored mild, 22 moderate, and 7 severe.

### Covariates of Psychological Distress

As shown in Table 2, the univariable logistic regression analysis showed that neither anxiety nor depression were associated with age, education, sexual orientation, migration background, years since HIV diagnosis, engaging in HIV care, HIV viral load, and STI diagnosis. Higher numbers of sexual partners and engaging in group sex were both associated with lower odds of anxiety and depression. Engaging in chemsex was associated with lower odds of depression on univariable analysis. Additionally, neither chemsex nor polydrug use was associated with anxiety.

**Table 2:** Univariable and multivariable logistic regressions assessing the associations between variables on anxiety and depression in gbMSM with HIV in the Netherlands (N = 359)

Variables	Potential anxiety disorder				Potential clinical depression			
	Univariable		Multivarable		Univariable		Multivatiable	
	OR	(95% CI)	aOR	(95% CI)	OR	(95% CI)	aOR	(95% CI)
Age								
< 25	1	–			1	–		
25–39	1.05	(0.35–3.12)			4.29	(0.53–34.62)		
> 40	0.63	(0.22–1.80)			4.22	(0.54–32.72)		
Education								
Second level or lower	1	–			1	–		
Third level or postgrad	0.69	(0.42–1.16)			0.85	(0.47–1.52)		
Sexual orientation								
Gay	1	–			1	–		
Bisexual/Other	1.75	(0.64–4.76)			1.71	(0.57–5.05)		
Migration background								
Dutch	1	–			1	–		
Western, non-Dutch	1.30	(0.47–1.22)			1.58	(0.54–4.63)		
Non-Western, non-Dutch	**2.35**	(1.22–4.55)			1.66	(0.79–3.48)		
HIV care								
Yes	1	–			1	–		
No	2.47	(0.48–12.48)			2.01	(0.36–11.27)		
Years since HIV diagnosis								
< 5 years (recent infection)	1	–			1	–	1	–
6–25 years (post ARVs)	0.49	(0.22–1.07)			1.35	(0.50–3.62)	2.37	(0.94–5.98)
> 25 years (pre ARVs)	0.72	(0.37–1.38)			1.89	(0.79–4.52)	0.84	(0.18–3.83)
Viral load								
Undetectable	1	–			1	–		
Detectable or unknown	0.81	(0.33–1.97)			0.73	(0.28–1.94)		
Number of sexual partners*								
0–1	1	–	1	–	1	–	1	–
2–4	0.61	(0.30–1.25)	0.53	(0.25–1.10)	**0.45**	(0.20–0.98)	0.48	(0.21–1.12)
5–10	0.45	(0.21–1.00)	0.35	(0.15–0.81)	**0.30**	(0.12–0.76)	0.29	(0.10–0.81)
> 10	**0.33**	(0.16–0.66)	0.23	(0.10–0.49)	**0.34**	(0.16–0.71)	0.28	(0.11–0.71)
Sexually transmitted infections*								
No	1	–	1	–	1	–	1	–
Yes	1.15	(0.68–1.92)	1.92	(1.05–3.49)	1.06	(0.59–1.90)	2.24	(1.08–4.64)
Group sex*								
No	1	–			1	0		
Yes	**0.56**	(0.34–0.91)			**0.49**	(0.28–0.87)		
Polydrug use*								
No	1	–			**1**	0		
Yes	0.62	(0.38–1.02)			**0.42**	(0.23–0.75)		
Chemsex** (8-chems)**								
No	1	–			1	–	1	–
Yes	0.66	(0.40–1.09)			**0.40**	(0.22–0.71)	0.45	(0.23–0.85)

*aOR* adjusted odds ratio, *ARVs* anti-retro viral medications, *CI* confidence interval, *gbMSM* gay, bisexual and other men who have sex with men, *HIV* human immunodeficiency virus

In bold if corresponding P-value < 0.05

*In the previous 6 months

**8 Chems: mephedrone, methamphetamine, GHB/GBL, cocaine, ketamine, speed, ecstasy/MDMA and/or other designer drugs

In multivariable logistic regression analysis, the number of sexual partners and STI diagnosis were the only variables associated with anxiety. Having ten or more sexual partners was associated with lower odds for anxiety compared to participants with fewer partners (aORs < 0.23, CI 0.10–0.49). Previous STI diagnoses increased the odds of anxiety (aOR 1.92, CI 1.05–3.49).

STI diagnosis was again was associated with increased odds of depression (aOR 2.24, CI 1.08–4.64). Importantly, chemsex was associated with lower odds of depression (aOR 0.45, CI 0.23–0.85). In addition, we checked the variables in the multivariate model for collinearity and did not find any, as indicated by tolerance coefficients between 0.308 and 0.882 (below 0.1 indicates possible collinearity), and variance inflation factors between 1.044 and 3.250 (above 5 indicates possible collinearity).

## Discussion

This study aimed to better understand the relationship between chemsex, anxiety and depression in gbMSM living with HIV. We found that engaging in chemsex, a behaviour traditionally thought of as high risk, was not associated with higher rates of anxiety and depression. Surprisingly, we found that engaging in chemsex was negatively associated with depression amongst gbMSM living with HIV in the Netherlands, such that people engaging in chemsex actually were less likely to qualify as potentially clinically depressed. Our findings contrast with the findings of previous research that has shown increased rates of psychological distress among gbMSM engaging in chemsex [5–8, 21]. It has however been posited that not all chemsex is harmful [22]. Contemporary literature on the phenomenon often divides chemsex into two distinct categories, problematic and non-problematic [23]. Problematic chemsex has been described as chemsex leading to negative consequences impacting health and wellbeing either through recurrent STIs, negative social outcomes (e.g. job loss, relationship issues, financial distress) adverse mental health impact or addiction [21]. Non-problematic chemsex lacks these negative outcomes [21]. It should also be noted that the findings from this research do not stand alone. Previous Australian research looked relationships between mental health, sexual risk behaviour and drug use, found that men who used drugs including amyl nitrates, cocaine and GHB were less likely to show signs of depression [24]. This study also found no association between anxiety and this type of illicit drug use [24]. Our study adds to previous research showing that chemsex is not always associated with increased rates of anxiety or depression, extending current evidence to include findings from gbMSM living with HIV who may face unique challenges related to their mental health.

Harm reduction strategies have been identified as a method of preventing chemsex from becoming problematic and resulting in adverse outcomes [25]. A previous study in the Netherlands highlighted that sexual health services are well equipped to deal with chemsex, with 71% of sexual health nurses surveyed indicating that they address chemsex regularly or always during routine consultations with gbMSM. This allowed for the timely identification of problematic chemsex use and referrals to specialised chemsex-related healthcare [26]. The negative association may be related to the fact that over 96% (n = 345) of participants were engaged in HIV care and may have access to these supports.

Our study also showed that another traditionally high-risk behaviour, increased numbers of sexual partners, was associated with experiencing less anxiety and depression. This may reflect that gbMSM living with HIV who have higher numbers of partners can experience increased *social connectedness* [3], which has been described as a fundamental human need and has been linked to better outcomes for both mental and physical health [3]. The association between higher numbers of partners and lower rates of psychological distress may also reflect reversed causality or bi-directional influences. More specifically, participants who score in the depressive range may be more likely to socially isolate and be less likely to seek out sexual partners, as lower libido has been associated with depression in gay men [27]. These participants may then also be less likely to come into contact or engage in chemsex.

### Strengths and Limitations

A strength of this study is that the participating gbMSM who live with HIV were not exclusively recruited via STI and HIV clinics; recruitment also took place on apps often used by gbMSM in the Netherlands. This reduces the risk of a selection bias and mitigates the risk of recruiting participants who are predominantly engaging with sexual health and HIV services. However, related to the inclusion of more sexually active men who use these apps. This selection bias may have increased the number of participants who engaged in higher-risk behaviours, including chemsex.

Another limitation of this research is the lack of data concerning when or why these drugs were being used. The frequency of use of the different drugs and issues like addiction and whether these participants had disclosed their chemsex use to a sexual health or HIV health professional and received education/harm reduction advice may have allowed for a greater understanding of how chemsex related to anxiety and depression among participants. This data would have contributed to a better understanding of the findings and may have allowed for identifying problematic versus non-problematic chemsex and how these relate to anxiety and depression among gbMSM living with HIV.

## Conclusion

Our findings highlight that chemsex was not associated with increased rates of anxiety and depression and was, surprisingly, associated with lower rates of depression among gbMSM living with HIV and unrelated with rates of anxiety. It is, however, important to note that the interplay between chemsex and mental health is complex, as the variation in findings of previous research shows. Findings suggest that for the participating gbMSM living with HIV chemsex use may not have been problematic when focusing on anxiety and depression. It is however, possible participants experienced other negative psychosocial impacts not measured in this study. Further research would benefit from a more comprehensive collection of data regarding chemsex to allow for a thorough interrogation of how methods of consumption, frequency of use and other associated aspects of chemsex may or may not be associated with mental health outcomes among gbMSM living with HIV and engaging in chemsex.

## Data Availability

The data supporting this study’s findings are available from the corresponding author on request.
